# In-Hospital Delays for Acute Stroke Treatment Delivery During the COVID-19 Pandemic

**DOI:** 10.1017/cjn.2020.170

**Published:** 2020-08-03

**Authors:** Aristeidis H. Katsanos, Danielle de Sa Boasquevisque, Mustafa Ahmed Al-Qarni, Mays Shawawrah, Rhonda McNicoll-Whiteman, Linda Gould, Brian Van Adel, Demetrios J. Sahlas, Kelvin Kuan Huei Ng, Kanjana Perera, Mukul Sharma, Wieslaw Oczkowski, Aleksandra Pikula, Ashkan Shoamanesh, Luciana Catanese

**Affiliations:** Division of Neurology, Hamilton General Hospital–Hamilton Health Sciences, McMaster University, Hamilton, Ontario, Canada; Population Health Research Institute, Hamilton, Ontario, Canada; Division of Neurology, University Hospital Network, University of Toronto, Hamilton, Ontario, Canada

**Keywords:** Caregiving, Thrombolysis

## Abstract

**Background:**

We investigated the impact of regionally imposed social and healthcare restrictions due to coronavirus disease 2019 (COVID-19) to the time metrics in the management of acute ischemic stroke patients admitted at the regional stroke referral site for Central South Ontario, Canada.

**Methods:**

We compared relevant time metrics between patients with acute ischemic stroke receiving intravenous tissue plasminogen activator (tPA) and/or endovascular thrombectomy (EVT) before and after the declared restrictions and state of emergency imposed in our region (March 17, 2020).

**Results:**

We identified a significant increase in the median door-to-CT times for patients receiving intravenous tPA (19 min, interquartile range (IQR): 14–27 min vs. 13 min, IQR: 9–17 min, *p* = 0.008) and/or EVT (20 min, IQR: 15–33 min vs. 11 min, IQR: 5–20 min, *p* = 0.035) after the start of social and healthcare restrictions in our region compared to the previous 12 months. For patients receiving intravenous tPA treatment, we also found a significant increase (*p* = 0.005) in the median door-to-needle time (61 min, IQR: 46–72 min vs. 37 min, IQR: 30–50 min). No delays in the time from symptom onset to hospital presentation were uncovered for patients receiving tPA and/or endovascular reperfusion treatments in the first 1.5 months after the establishment of regional and institutional restrictions due to the COVID-19 pandemic.

**Conclusion:**

We detected an increase in our institutional time to treatment metrics for acute ischemic stroke patients receiving tPA and/or endovascular reperfusion therapies, related to delays from hospital presentation to the acquisition of cranial CT imaging for both tPA- and EVT-treated patients, and an added delay to treatment with tPA.

## RÉSUMÉ

**Délais dans le traitement en milieu hospitalier des AVC aigus dans le contexte de la pandémie de COVID-19.**

**Contexte:**

Nous nous sommes penchés, dans le contexte de la pandémie de COVID-19, sur l’impact de restrictions régionales imposées dans le domaine social et dans les soins de santé sur les délais de prise en charge de patients victimes d’un AVC aigu. À noter que ces patients ont été admis dans un centre régional de traitement des AVC situé dans le centre-ouest de l’Ontario (Canada).

**Méthodes:**

Nous avons comparé entre eux les délais de prise en charge de patients ayant bénéficié d’activateurs tissulaires du plasminogène par intraveineuse (tPA) et/ou d’une procédure de thrombectomie endovasculaire (TE) avant et après la mise sur pied de restrictions et l’imposition d’un état d’urgence sanitaire dans notre région (17 mars 2020).

**Résultats:**

Après la mise sur pied de ces restrictions, nous avons identifié, par rapport aux 12 mois précédent, une augmentation notable des délais médians entre l’arrivée à l’hôpital et un examen de tomodensitométrie dans le cas de patients bénéficiant de tPA (19 minutes, EI : 14–27 minutes contre 13 minutes, EI : 9–17 minutes ; *p* = 0,008) et/ou d’une procédure de TE (20 minutes, EI : 15–33 minutes contre 11 minutes, EI : 5–20 minutes ; *p* = 0,035). Pour ce qui est des patients bénéficiant de tPA, nous avons également observé une augmentation importante (*p* = 0,005) des délais médians entre leur arrivée à l’hôpital et l’injection d’un traitement (61 minutes, EI : 46–72 minutes contre 37 minutes, EI : 30–50 minutes). Enfin, dans le premier mois et demi suivant la mise sur pied des restrictions régionales et institutionnelles attribuables à la pandémie de COVID-19, aucun délai supplémentaire entre l’apparition des premiers symptômes d’un AVC et l’arrivée à l’hôpital n’a été remarqué pour des patients bénéficiant de tPA et/ou d’une procédure de TE.

**Conclusion:**

En somme, nous avons détecté une augmentation de nos délais de traitement dans le cas de patients victimes d’un AVC aigu ayant bénéficié de tPA et/ou d’une procédure de TE. Cela peut être attribué à une augmentation des délais de présentation à l’hôpital mais aussi à des délais dans l’obtention d’images de tomodensitométrie pour des patients traités avec des tPA et une procédure de TE, sans compter des délais accrus pour bénéficier d’un traitement de tPA.

## Introduction

The severe acute respiratory syndrome coronavirus 2 (SARS-CoV-2), originally emerging in Wuhan, has quickly spread worldwide and coronavirus disease 2019 (COVID-19) was declared as a pandemic outbreak on March 11, 2020.^1^ Reports have emerged globally on the impact of the COVID-19 pandemic on the management of stroke patients. Stroke experts and international organizations have highlighted the need to preserve the best standards and comprehensiveness of care at all stages during the COVID-19 pandemic outbreak.^2,3^ Reports of declining stroke admission volumes and delays in hospital presentation, which have resulted in decreased systematic and endovascular reperfusion treatments, due to presumed patient fears, as well as the social and logistical barriers imposed by community and healthcare preventive measures, are accumulating.^4-7^

In the present report, we investigate the impact of regionally imposed social and healthcare restrictions to the quality of care time metrics in the hyperacute management of patients presenting with acute ischemic strokes to a large comprehensive stroke center in Ontario, Canada.

## Methods

We performed a retrospective review on the time metrics of consecutive stroke patients admitted or transferred for acute stroke treatment to the Hamilton General Hospital, Hamilton Health Sciences between March 1, 2019 and April 30, 2020. The Hamilton General Hospital is the regional referral comprehensive stroke center for a population of 2.2 million people in Central South Ontario, Canada and cares for over 1500 patients with stroke and threatened stroke annually.^8^

For the aforementioned time period, we retrospectively searched hospital databases and records to obtain the total numbers of acute ischemic stroke patients receiving systemic and/or endovascular reperfusion therapies with the relevant time metrics. Additional informations for patients receiving endovascular thrombectomy (EVT) were obtained from an established national EVT registry (OPTIMISE) that focuses on improving the quality of management of patients receiving EVT for acute ischemic stroke. The primary outcome of interest was the time from hospital admission to treatment initiation, using the door-to-needle and door-to-groin puncture times for patients receiving treatment with intravenous tissue plasminogen activator (tPA) and/ or EVT, respectively.

For acute ischemic stroke patients receiving intravenous tPA treatment, we extracted additional data on the time from symptom onset to hospital presentation (onset-to-door time), time spent in triage at the emergency room (ER triage), time from the presentation at our institution to the first neuroimaging acquisition (door-to-CT time), time from first neuroimaging acquisition to the start of the tPA bolus (CT-to-needle time), and the total time from symptom onset to tPA bolus administration (onset-to-treatment time). For patients receiving EVT, we extracted data on the time from arrival at our institution to the initiation of EVT (door-to-groin puncture), the time from angiography suite arrival to groin puncture time, and the time from stroke symptoms onset to groin puncture (onset-to-groin puncture time). Finally, we also reported the time from hospital arrival to the last angiographic run establishing any degree of reperfusion (door-to-recanalization). For patients transferred for EVT to our center from a primary stroke care center, we additionally obtained data on the time that the patient stayed in the place of the first arrival until the initiation of transfer to our institution (door-in-to-door-out time).

For the primary analyzes, we compared all relevant time metrics between patients with acute ischemic stroke receiving intravenous tPA and/or EVT prior to the declared lockdown restrictions and state of emergency imposed in Ontario (between March 17, 2020 and April 30, 2020) and acute ischemic stroke patients receiving intravenous tPA and/or EVT between March 1, 2019 and March 16, 2020. Following the declaration of government-imposed social distancing on March 17, 2020, our institution instantly activated staff redeployment, physical distancing, screening at all hospital entrances, and the default use of personal protective equipment for all new admissions to the emergency department.

As sensitivity analyzes, we compared the aforementioned time metrics between acute ischemic stroke patients presenting between March 17, 2020 and April 30, 2020 and ischemic stroke patients treated during the respective time period the previous year (March 17, 2019–April 30, 2019). Additionally, using box plots, we present a biweekly temporal overview of door-to-CT time, which is a common metric for both tPA- and EVT-treated patients, from December, 2019 to April, 2020. Dichotomous variables were presented as percentages, while continuous variables were summarized using median values and corresponding interquartile ranges (IQRs). Dichotomous variables were analyzed using Pearson’s chi-square test and continuous variables with the Mann–Whitney U test. Analyzes were performed with the Stata Statistical Software Release 13 for Windows (College Station, TX, StataCorp LP).

## Results

**Figure 1:**
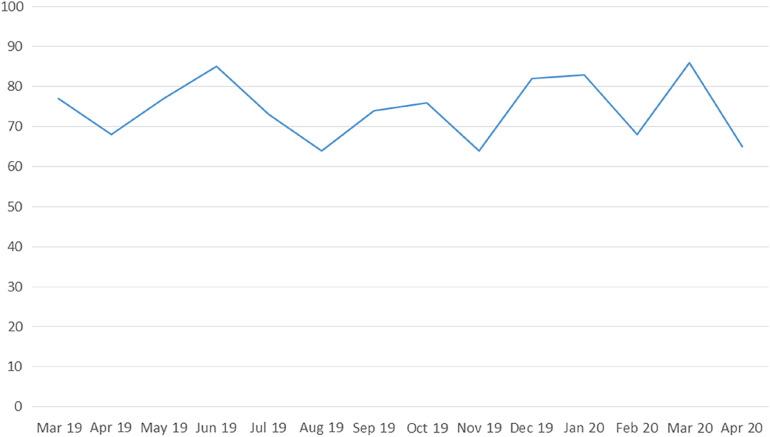
Overview of monthly stroke admissions over the period March, 2019–April, 2020 in our institution.

**Figure 2:**
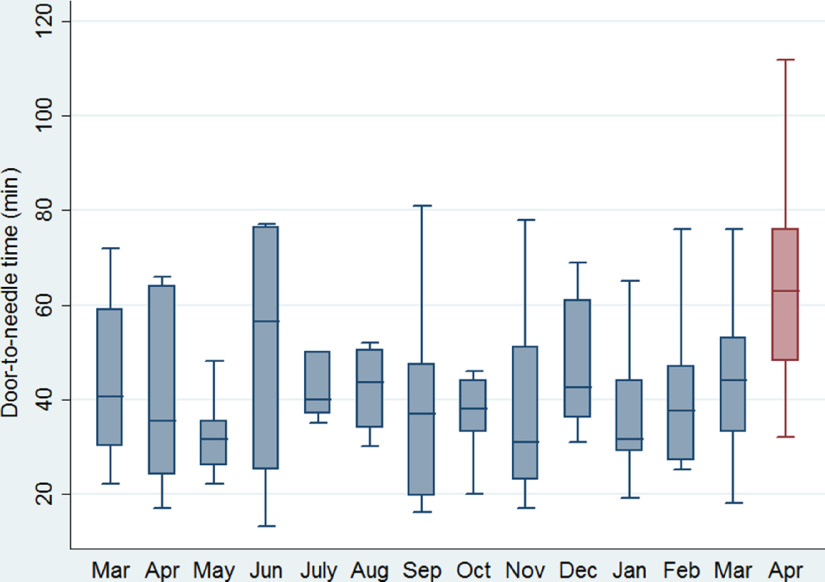
Box plots presenting a monthly overview of the timing from hospital presentation to the initiation of intravenous tPA for acute ischemic stroke patients receiving treatment with intravenous thrombolysis in our institution.

**Figure 3:**
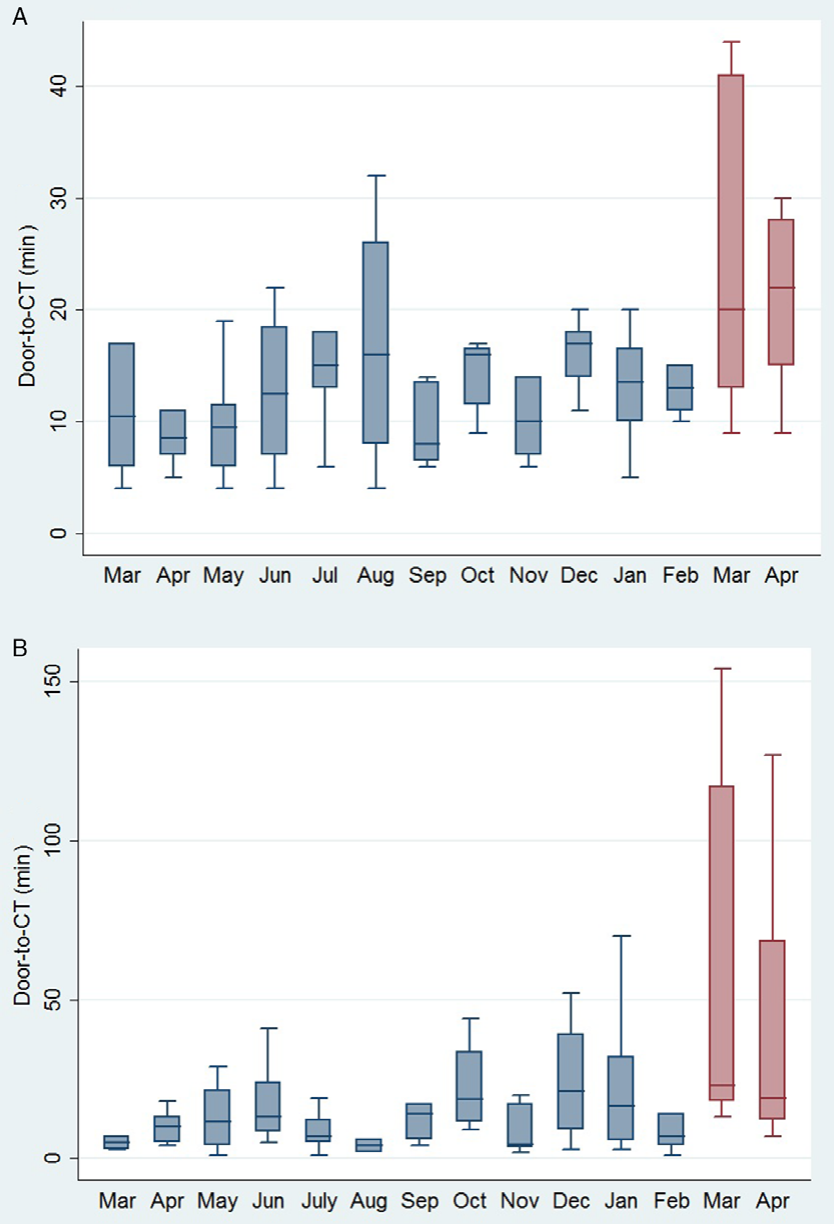
Box plots presenting a monthly overview of the timing from hospital presentation to computed tomography acquisition for acute ischemic stroke patients receiving treatment with (A) intravenous tPA and/or (B) EVT in our institution.

The monthly number of stroke admissions from March 1, 2019 to April 30, 2020 is shown in Figure 1. Stroke admissions seem to have a variation over this time period, without any obvious trend being uncovered after March, 2020. Patients receiving tPA treatment after the official declaration of social and healthcare restrictions in our region were found to have a significant increase (*p* = 0.005) in the median door-to-needle time (61 min, IQR: 46–72 min) compared to patients receiving tPA treatment the previous 12 months (37 min, IQR 30–50 min; Figure 2). This finding was relevant to increases in both door-to-CT times (19 min, IQR: 14–27 min vs. 13 min, IQR: 9–17 min, *p* = 0.008; Figure 3A) and CT-to-needle times (38 min, IQR: 26–46 min vs. 24 min, IQR: 17–33, *p* = 0.023). No significant differences in onset-to-door (*p* = 0.480), ER triage (*p* = 0.888), and onset-to-treatment times (*p* = 0.394) were found between patients receiving tPA treatment before and after the start of social distancing measures (Table 1). In sensitivity analyzes (Table 2), door-to-needle and door-to-CT times were again found to be significantly prolonged for acute ischemic stroke patients receiving tPA treatment between March 17, 2020 and April 30, 2020 compared to acute ischemic stroke patients receiving tPA treatment during the same time period a year ago (March 17, 2019–April 30, 2019).

**Table 1:** Time metrics in the treatment of acute ischemic stroke patients before and after the declared regional social and healthcare restrictions

	After SHR	Before SHR	*p*-value
Intravenous thrombolysis with tPA			
Number of patients	12	97	
Age (years, median, IQR)	68 (56–78)	74 (64–85)	0.220
NIHSS score on admission (median, IQR)	8 (4–12)	12 (8–19)	0.010
Onset-to-door time (min, median, IQR)	55 (45–74)	64 (44–92)	0.480
ER triage time (min, median, IQR)	5 (3–7.5)	4 (3–6)	0.888
Door-to-CT time (min, median, IQR)	19 (14–27)	13 (9–17)	0.008
CT-to-needle time (min, median, IQR)	38 (26–46)	24 (17–33)	0.023
Door-to-needle time (min, median, IQR)	61 (46–72)	37 (30–50)	0.005
Onset-to-treatment time (min, median, IQR)	121 (87–183)	104 (77–147)	0.394
Endovascular thrombectomy			
Number of patients	18	154	
Age (years, median, IQR)	68 (62–84)	74 (62–83)	0.681
NIHSS score on admission (median, IQR)	17 (13–21)	18 (14–22)	0.602
Transfer from primary stroke center (%)	55.5%	63.6%	0.502
tPA pretreatment (%)	70.5%	45.8%	0.052
Door-in-to-door-out time (min, median, IQR)*	94 (79–116)	91 (79–110)	0.656
Onset-to-door time (min, median, IQR)	154 (90–226)	235 (145–415)	0.037
Door-to-CT time (min, median, IQR)	20 (15–33)	11 (5–20)	0.035
Angiography suite arrival to groin puncture time (min, median, IQR)	19 (16–22)	16 (13–20)	0.022
Door-to-groin puncture time (min, median, IQR)	60 (33–110)	43 (29–82)	0.391
Onset-to-groin puncture time (min, median, IQR)	238 (180–275)	293 (201–453)	0.164
Door-to-recanalization (min, median, IQR)	117 (62–139)	77 (58–123)	0.348

ER = emergency room; IQR = interquartile range; NIHSS = National Institutes of Health Stroke Scale; SHR = social and healthcare restrictions; tPA = tissue plasminogen activator.

* For patients transferred from a primary center for endovascular thrombectomy.

**Table 2:** Time metrics in the treatment of acute ischemic stroke patients presenting after the declared regional lockdown restrictions and patients presenting the same time period a year ago

	March 17, 2020–April 30, 2020	March 17, 2019–April 30, 2019	*p*-value
Intravenous thrombolysis with tPA			
Number of patients	12	8	
Age (years, median, IQR)	68 (56–78)	69 (54–88)	0.938
NIHSS score on admission (median, IQR)	8 (4–12)	10 (9–15)	0.114
Onset-to-door time (min, median, IQR)	55 (45–74)	65 (54–86)	0.315
ER triage time (min, median, IQR)	5 (3–7.5)	5 (3.5–6)	0.877
Door-to-CT time (min, median, IQR)	19 (14–27)	7.5 (5.5–10)	0.004
CT-to-needle time (min, median, IQR)	38 (26–46)	27 (17–37)	0.280
Door-to-needle time (min, median, IQR)	61 (46–72)	35 (23–52)	0.005
Onset-to-treatment time (min, median, IQR)	121 (87–183)	99 (81–138)	0.335
Endovascular thrombectomy			
Number of patients	18	11	
Age (years, median, IQR)	68 (62–84)	74 (59–78)	0.982
NIHSS score on admission (median, IQR)	17 (13–21)	18 (9–19)	0.347
Transfer from primary stroke center (%)	55.5%	63.6%	0.668
tPA pretreatment (%)	70.5%	54.5%	0.387
Door-in-to-door-out time (min, median, IQR)*	94 (79–116)	81 (77–93)	0.182
Onset-to-door time (min, median, IQR)	154 (90–226)	262 (131–415)	0.121
Door-to-CT time (min, median, IQR)	20 (15–33)	10 (5–18)	0.044
Angiography suite arrival to groin puncture time (min, median, IQR)	19 (16–22)	15 (13–20)	0.092
Door-to-groin puncture time (min, median, IQR)	60 (33–110)	50 (31–114)	0.962
Onset-to-groin puncture time (min, median, IQR)	238 (180–275)	320 (188–446)	0.281
Door-to-recanalization (min, median, IQR)	117 (62–139)	68 (52–98)	0.405

ER = emergency room; IQR = interquartile range; NIHSS = National Institutes of Health Stroke Scale; tPA = tissue plasminogen activator.

For acute ischemic stroke patients treated with EVT, we also detected a significant increase in door-to-CT (20 min, IQR: 15–33 min vs. 11 min, IQR: 5–20 min, *p* = 0.035; Figure 3B) and angiography suite arrival-to-groin puncture times (19 min, IQR: 16–22 min vs. 16 min, IQR: 13–20, *p* = 0.022) following the official implementation of social and healthcare restrictions in Ontario. Interestingly, the median time from symptom onset to admission in our institution (onset-to-door time) was shorter for patients presenting after March 17, 2020 (154 min, IQR: 90–226 min vs. 235 min, IQR: 145–415 min, *p* = 0.037). Although the median door-to-recanalization time was found to be prolonged (117 min, IQR: 62–139 vs. 77 min, IQR 58–123 min), this difference did not reach statistical significance (*p* = 0.348). When restricting the comparison cohort to patients with acute ischemic stroke receiving EVT during the corresponding time interval a year ago (March 17, 2019–April 30, 2019) only a significant difference in door-to-CT times was uncovered (*p* = 0.044; Table 2). We found no difference in the rate of transfers from primary stroke care centers or tPA pretreatment among acute ischemic stroke patients receiving EVT treatment before and after the establishment of regional social and healthcare restrictions. No delays in primary stroke care centers (door-in-to-door-out time) or in the initiation of EVT in our institution were uncovered in both primary (Table 1) and sensitivity analyzes (Table 2).

## Discussion

**Figure 4:**
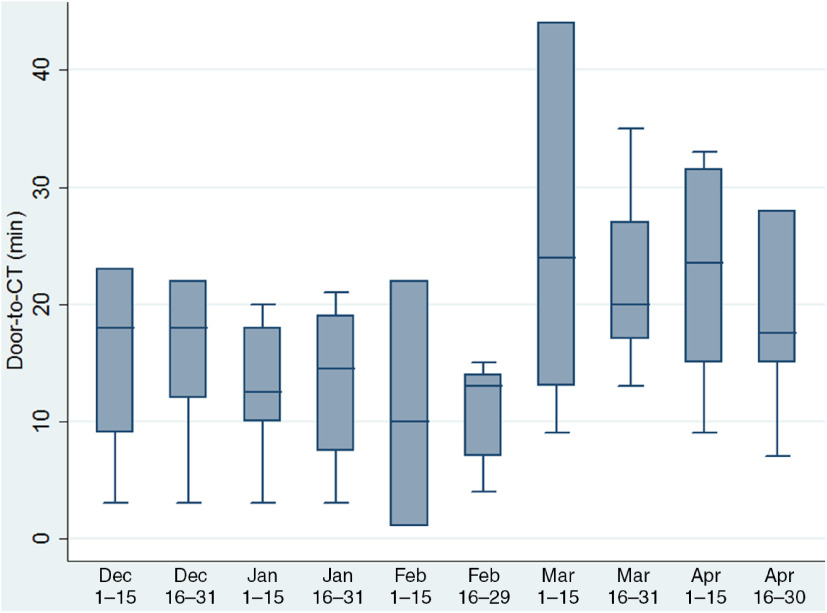
Box plots presenting a biweekly temporal overview from December, 2019 to April, 2020 on the timing from hospital presentation to computed tomography acquisition for acute ischemic stroke patients receiving treatment with intravenous tPA and/or EVT in our institution.

We identified an increase in our institutional in-hospital time to treatment metrics on the administration of both tPA and endovascular reperfusion therapies for patients with acute ischemic stroke presenting after the official establishment of social and healthcare restrictions in our region. These delays were primarily related to an increased time from hospital presentation to the acquisition of CT scan for both tPA- and EVT-treated patients and an increased time to tPA administration from CT scan acquisition. Increase in door-to-CT times was already noticed from the first days of March (Figure 4), highlighting the impact of COVID-19 pandemic in stroke metrics even before the official announcement of public and healthcare restrictions.

This report raises significant concerns for increased in-hospital delays for acute stroke treatment delivery following social distancing and healthcare restriction measures related to the COVID-19 pandemic. Aiming to protect healthcare workers and reduce the impact of the COVID-19 surge on mortality and morbidity, stroke centers have been guided to implement additional precautions in the first contact with acute stroke patients, assuming by default that every incoming stroke patient is potentially infected with COVID-19.^9^ Time delays are potentially related to initial screening of respiratory or gastrointestinal symptoms on the first encounter at the emergency department triage and by the stroke team, the application of personal protective equipment, isolation precautions of patients screening COVID-19 positive, the extra caution at every step within the acute stroke response pathway, and during the completion of the CT imaging, implementation of staff screening prior to entry to the hospital and changes in staff access at hospital entryways. Except for the preventive measures delaying prompt stroke care delivery in the emergency setting, concerns have been expressed for decreased stroke team stamina due to understaffing and extended shifts, as results of prophylactic staff quarantine or COVID-19 illness. The use of personal protective equipment has additionally been associated with decreased healthcare personnel endurance due to discomfort and headache.^10^

Our report is in line with a recent publication from a Spanish regional stroke care system also highlighting an increased door-to-needle time for tPA patients in the COVID-19 era.^11^ However, our report is the first to date reporting the presence of in-hospital delays for both tPA- and EVT-treated patients in the common pathway from hospital presentation to CT acquisition. There are several limitations that need to be acknowledged. First, we extracted and report aggregate patient data on time metrics, while individual patient characteristics regarding stroke severity, past medical history, or imaging findings were not assessed. Second, we have no data on the functional outcomes following acute stroke treatment administration. Therefore, we cannot assess the potential impact of healthcare and social restrictions on the patient outcomes. Third, the number of stroke patients treated with either intravenous tPA and/or EVT over the 1.5 months after the implication of social and healthcare restrictions is limited and, therefore, the lack of statistical differences in some of the time metrics could be related to low power rather than the lack of true differences. Fourth, contrary to reports from other institutions in other regions of the world^7^, we did not uncover any delays in the presentation of patients to the hospital or in the total time from symptom onset to treatment delivery. It is uncertain whether differences in individual patient demographics, stroke severity, and/or a reduction in emergency medical services (EMS) volumes during COVID-19 measures could have contributed to these observations. Additionally, social restrictions urged family members to spend significantly more time together at home, which might lead to timely recognition of stroke symptoms and prompt EMS notification. Therefore, according to our findings, we suggest that institutional activated restrictions and preventive measures had a significant impact on the in-hospital acute stroke management, while we were not able to uncover any relevant impact of social distancing and overall public apprehension on the acute stroke prehospital pathway. Our data derive from a single regional comprehensive stroke center in Central South Ontario and thus might not be relevant to other institutions or regions. The disparity of our findings with previous reports highlights the need for intensive quality monitoring of stroke care delivery during the COVID-19 pandemic.

Our single-center experience suggests that healthcare institutional restrictions imposed in response to the COVID-19 pandemic have had a negative impact on acute ischemic stroke care time metrics known to predict stroke-related clinical outcomes. To preserve quality in acute stroke care patient access and outcomes, while decreasing potential COVID-19 exposure to patients and healthcare providers, institutions should consider simulation training programs to prepare their medical teams for protected code strokes during the COVID-19 pandemic.^12,13^
